# The complete mitochondrial genome of *Paragabara curvicornuta* (Lepidoptera: Noctuoidea: Eribidae)

**DOI:** 10.1080/23802359.2017.1422406

**Published:** 2018-01-05

**Authors:** Zi-Shu Chen, Zhi Xu, Li Ma, Xing Wang

**Affiliations:** a College of Plant Protection, Hunan Agricultural University, Changsha, Hunan, China;; b Hunan Provincial Key Laboratory for Biology and Control of Plant Diseases and Insect Pests, Hunan Agricultural University, Changsha, Hunan, China

**Keywords:** *Paragabara curvicornuta*, mitochondrial genome, evolutionary position

## Abstract

In this study, the complete mitochondrial genome (mitogenome) of *Paragabara curvicornuta* was sequenced for the first time by traditional PCR amplification and primer walking methods. As a circular DNA molecule, the entire mitogenome is 15,532 bp in length (GeneBank accession no. KT362742), and consists of 13 protein-coding genes (PCGs), 22 transfer RNA (tRNA) genes, two ribosomal RNA (rRNA) genes and an AT-rich region. The nucleotide composition is A (39.9%), C (12.0%), G (7.6%), and T (40.4%). Based onthe amino acid sequences of 13 PCGs from 43 noctuoid species as ingroups and two drepanoid species as outgroups, the phylogenetic trees was constructed. The phylogenetic relationships of the families among Noctuoidea are (Notodontidae, (Erebidae, (Nolidae, Noctuidae)). However, the phylogenetic position of *P. curvicornuta* is still unclear in the family Erebidae that is a monophyletic clade strongly supported by the bootstrap value of 100%.

As a Hypeninae species, *Paragabara curvicornuta* Kononenko, Han & Matov, 2010 is widely distributed in Russian Far East, Japan, Korea and Northeastern China (Kononenko et al. 2010). Its phylogenetic relationship among the superfamily Noctuoidea is debated (Zahiri et al. 2011; Mitter et al. 2017). In July 2014, the *P. curvicornuta* larvae on *Glycine max* (Linn.) Merr. were collected from the experimental fields in the campus of Hunan Agricultural University (28° 11'03″N, 113°04'26″E, elevation of 47 m), and the specimens of all the different stages (eggs, larvae, pupae and adults) were obtained. The whole genomic DNA was extracted from a pupa by the standard phenol-chloroform extraction procedure. All the specimens and the genomic DNA were deposited in Insect Museum of Hunan Agricultural University, Changsha City, Hunan Province, China.

The complete mitochondrial genome (mitogenome) of *P. curvicornuta* was sequenced for the first time by primer walking methods with the reported primers (Gu et al. 2016), and compared with the mitogenomes of noctuoid species sequenced. The sequences of *P. curvicornuta* were proof-read and assembled using the program Geneious 8.12 (Kearse et al. 2012). The mitogenome was annotated using the ORF finder (https://www.ncbi.nlm.nih.gov/orffinder) and the nucleotide blasts (https://blast.ncbi.nlm.nih.gov/Blast.cgi) on the NCBI database. To construct the phylogenetic relationship within the superfamily Noctuoidea, the complete mitogenomes of 42 noctuoid species, which plus *P. curvitornuta* as ingroups, and 2 drepanoid species as outgroups were downloaded. The maximum likelihood (ML) was performed via the software IQTree 1.5.5 (http://www.iqtree.org/) (Lam-Tung et al. 2015) with the Number of Bootstrap Replications as 1000.

The complete mitogenome of *P. curvicornuta* is a circular DNA molecule of 15,532 bp in length (GeneBank accession no. KT362742), and consists of 13 protein-coding genes (PCGs), 22 transfer RNA (tRNA) genes, two ribosomal RNA (rRNA) genes and an AT-rich region. In which, 23 genes are transcribed on the J strand and the remaining 14 are transcribed on the N strand. The nucleotide composition is A (39.9%), C (12.0%), G (7.6%), and T (40.4%), and the AT nucleotide content is 80.4%. There were 273 bp intergenic nucleotides that were dispersed in 16 pairs of neighbouring genes with their length varying from 1 to 52 bp. The length of the A + T-rich region that was located between *rrnS* and the *trnM* was 364 bp. The phylogenetic tree based on the putative amino acids sequences from all 13 PCGs with excluding the stop codons is shown in Figure 1. The relationship within this superfamily Noctuoidea that is a monophyletic clade supported by the bootstrap value of 100% is (Notodontidae, (Erebidae, (Nolidae, Noctuidae)), which is consistent with previously reported results (Regier et al. 2017). Furthermore, the family Erebidae as a monophyletic clade is strongly supported by the bootstrap value of 100%. But, the phylogenetic position of *P. curvicornuta* is still unclear in the family Erebidae, and more data are necessary for further study.

**Figure 1.**
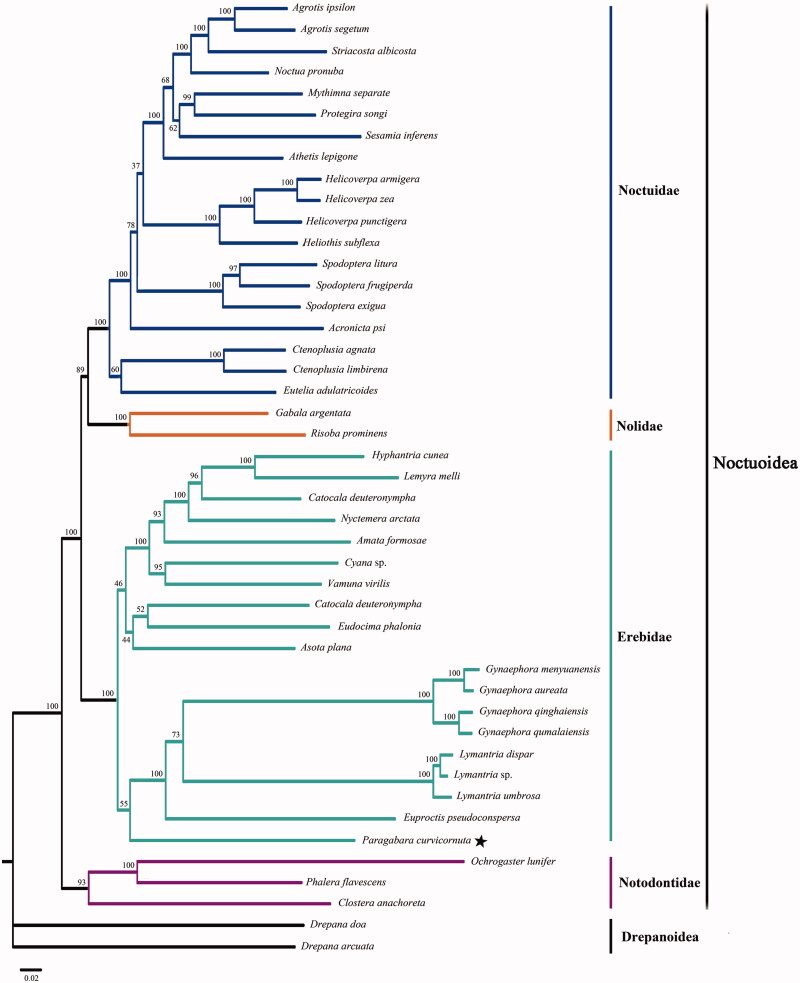
Maximum-likelihood phylogram constructed using 13 PCGs of mitogenomes. Numbers above each node indicates the ML bootstrap support values. All the species’ accession numbers in this study are listed as below: *Acronicta psi* KJ508060, *Agrotis ipsilon* NC_022185, *Agrotis segetum* NC_022689, *Amata formosae* NC_021416, *Asota plana* KJ173908, *Athetis lepigone* NC_036057, *Callimorpha dominula* NC_027094, *Catocala deuteronympha* KJ432280, *Clostera anachoreta* NC_034740, *Ctenoplusia agnate* NC_021410, *Ctenoplusia limbirena* NC_025760, *Cyana* sp KM244679, *Drepana arcuata* KJ508053, *Drepana doa* KJ508058, *Eudocima phalonia* NC_032382, *Euproctis pseudoconspersa* NC_027145, *Eutelia adulatricoides* NC_026840, *Gabala argentata* NC_026842, *Gynaephora aureata* NC_029162, *Gynaephora menyuanensis* NC_020342, *Gynaephora qinghaiensis* NC_029163, *Gynaephora qumalaiensis* NC_029164, *Helicoverpa armigera* NC_014668, *Helicoverpa punctigera* NC_023791, *Helicoverpa zea* NC_030370, *Heliothis subflexa* NC_028539, *Hyphantria cunea* NC_014058, *Lemyra melli* NC_026692, *Lymantria dispar* NC_012893, *Lymantria* sp. KY923068, *Lymantria umbrosa* NC_035627, *Mythimna separate* NC_023118, *Noctua pronuba* KJ508057, *Nyctemera arctata* KM244681, *Ochrogaster lunifer* NC_011128, *Phalera flavescens* NC_016067, *Protegira songi* NC_034938, *Risoba prominens* NC_026841, *Sesamia inferens* NC_015835, *Spodoptera exigua* NC_019622, *Spodoptera frugiperda* NC_027836, *Spodoptera litura* NC_022676, *Striacosta albicosta* NC_025774, *Vamuna virilis* NC_026844.
